# Adherence to Antibiotic Prophylaxis in Children with Vesicoureteral Reflux

**DOI:** 10.1155/2011/134127

**Published:** 2011-03-27

**Authors:** Esequiel Rodriguez, Dana A. Weiss, Hillary L. Copp

**Affiliations:** UCSF Department of Urology, University of California, 400 Parnassus Ave, A633 San Francisco, CA 94143-0738, USA

## Abstract

Vesicoureteral reflux (VUR) affects approximately 1% of children and may predispose a child with a bladder infection to develop pyelonephritis and renal scarring. To prevent these potential sequelae, one accepted treatment option for VUR includes low-dose continuous antibiotic prophylaxis (CAP) to maintain urine sterility until the condition resolves. Despite the widespread use of CAP, little data exists regarding adherence to long-term antibiotic therapy. Not only will poor adherence to CAP potentially preclude the intended benefit, but also nonadherence with antibiotic regimens may carry untoward effects including unnecessary treatment changes for presumed antibiotic failure, emergence of resistant organisms, and compromised clinical trial outcomes. We present an overview of medication adherence in children with VUR, discuss possible consequences of nonadherence to antibiotic prophylaxis, and suggest ways to improve adherence. We raise awareness of issues related to nonadherence relevant to healthcare providers, investigators, and the community.

## 1. Introduction

Vesicoureteral reflux (VUR), a common congenital abnormality, affects approximately 1% of infants and children. It may predispose a child with a bladder infection to the development of pyelonephritis, which may lead to renal scarring and hypertension [1]. To prevent these potential long-term sequelae, one accepted treatment option for persistent VUR includes low-dose continuous antibiotic prophylaxis (CAP). Since reflux has a 5–13% annual spontaneous resolution rate, CAP may be offered as a protective measure to maintain urine sterility until the condition resolves [2, 3]. Herein, the physician assumes the patient is taking the antibiotic as prescribed on a daily basis. 

Despite the widespread use of CAP, there is a paucity of data regarding patient adherence to long-term antibiotic therapy. The intended benefit of any medication will be completely achieved only if patients adhere to the prescribed treatment regimen. Not only will nonadherence to CAP potentially preclude the intended benefit, but also nonadherence with antibiotic regimens may carry potential untoward effects. For example, nonadhernence to antibiotics is associated with the emergence of resistant organisms [4, 5]. In addition, recurrent UTIs in nonadherent patients with VUR may be interpreted as treatment failure, and may instigate unnecessary treatment changes. Nonadherence may also compromise clinical trial outcomes and, consequently, treatment guidelines derived from them.

The aim of this paper is to provide a brief overview of medication adherence in children with VUR, discuss possible consequences of nonadherence to antibiotic prophylaxis, and suggest ways to improve adherence. Our goal is to raise awareness of issues related to nonadherence relevant to healthcare providers, investigators, and the community.

## 2. Adherence

The intended benefit of any prescribed medication will be fully achieved only if the patient adheres to the prescribed treatment closely. Adherence to a medication regimen is generally defined as the degree to which the medications taken reflect the prescribing healthcare provider's intention [6, 7]. While there is no consensus on what constitutes adequate adherence, an adherence rate of 80% or greater (antibiotic coverage for 80% of the year or more) is commonly used in the literature [8]. Similarly, there is no consensual gold standard method for measuring whether patients take the medication prescribed or not. Current methods used to measure compliance are best categorized as direct and indirect methods [8–10]. Direct methods include biologic blood markers, level of medicine or metabolite in blood or urine, and direct observation of ingestion. Indirect methods include patient/caregiver questionnaire, medication diary, medical record, pill counts, rates of prescription refills, and patient's clinical response. In general, direct methods more reliably predict adherence; however, they are more costly and time consuming. Many of the indirect methods are more practical; however, they have been shown to overestimate adherence [8–10]. Using a parent questionnaire, Smyth showed that 97% of parents reported giving prophylactic antibiotics every day; however, only 67% of urine samples tested positive for antibacterial substance [11]. In a similar study of children with VUR, in the group without recurrent urine infections 84% of parents reported that their children were taking antibiotics at 6 months but only 28% had a positive urine antibiotic activity test; at 1 year 57% reported their children were taking antibiotics but only 17% had a positive urine test [12]. It's important to note that even direct measuring methods with ultrasensitive blood or urine tests may not be entirely reflective of adherence. Many patients improve medication compliance just before seeing their healthcare provider [13]. In this scenario, blood or urine medication level only indicates that the medication was recently taken, but does not ascertain adherence in between visits.

Regardless of the method used to measure adherence, a common denominator among children with chronic diseases such as asthma, diabetes, HIV, and skin diseases, is that poor adherence is a ubiquitous phenomenon. In a quantitative review of children with chronic diseases, DiMatteo estimated that only about 70% of patients were adherent to treatment recommendations [14]. However, other studies have noted even lower rates, averaging about 50–60%, with decreasing compliance rates noted over time [15–18]. In a prospective study of children with asthma, adherence rates to inhaled corticosteroids decreased from 73% at 4 months to 59% at 12 months [19]. Even in clinical trials where patients often receive more attention and, by nature, may self-select for inherently motivated subjects, poor adherence rates are not uncommon. In the PENTA 5 randomized controlled trial evaluating HIV treatment in children, 25% of subjects were nonadherent with antiretroviral regimens [20].

Congruent with the poor adherence rates witnessed in children with other chronic diseases, adherence with prophylactic antibiotics in children with VUR is poor. Additionally, with time, adherence with long-term antibiotic decreases dramatically [10, 12]. Using large pharmacy claims databases in children with VUR, two recent studies showed poor prophylactic antibiotic adherence as defined by an adherence rate of less than 80%. In the first study, Hensle and colleagues reported a 17% adherence rate [21]. Similarly, in the second study, Copp et al. reported a 40% adherence rate [22]. These two recent reports substantiate previous findings where antibiotic adherence rates at one year among children with VUR were only 57% by parental report and 17% by measured urine levels of antibiotic activity [12].

## 3. Potential Consequences of Nonadherence

Omitting antibiotic doses may result in breakthrough UTIs, which in addition to the discomfort and inconvenience caused, may increase the risk for pyelonephritis and renal scarring [1]. Furthermore, recurrent UTI's in nonadherent patients with VUR may be interpreted as treatment failure, and may instigate unnecessary medication changes or additional interventions such as imaging studies and/or surgical correction of the underlying condition. Unnecessary tests and treatments, hospital admissions, and additional healthcare provider visits increase healthcare costs [23, 24]. Moreover, indirect costs and effects such as workdays missed by parents and school absences by children create a burden that is difficult to quantify. 

In addition to affecting the individual patient, poor adherence may also have local and global ramifications. Bacterial drug resistance is a growing problem and has become a major public health concern. For example, *E Coli*, which causes 70–90% of first febrile uncomplicated UTIs in children [25–28], is resistant to sulfonamides in up to 27% of cases in certain regions of the United States [29]. The emergence of drug resistant strains is more likely to occur in nonadherent patients, as subtherapeutic antibiotic levels have been shown to foster the development of resistant organisms [4, 5, 30].

Lastly, poor adherence may compromise clinical trial outcomes and, consequently, treatment recommendations derived from them. Four well-referenced contemporary randomized controlled studies in children with VUR question the benefit of antibiotic prophylaxis as UTI, pyelonephritis, and renal scaring were not statistically different among patients receiving versus not receiving CAP [30–33]. All four studies failed to account for adherence to the antibiotic treatment therapy. Since nonadherence has also been shown to be an important factor in the research setting [20], a possible explanation for these findings could simply be due to a lack of adherence to the daily antibiotic prophylaxis regimen. 

As investigators we cannot assume that children/parents are adherent to treatment. We must adequately account for and report adherence rates in our studies. Adherence estimates determined solely by the attending doctor should be discouraged as they have been shown to be inaccurate and overestimate true rates [34, 35]. In a prospective study measuring compliance with antibiotic prophylaxis in children with sickle cell anemia, adherence rate was low when measured by urine tests (56%) and parental interview (48%), but overestimated by analysis of medical records (89%) [34]. Given its complexity, to maximize accuracy, a combination of methods for measuring adherence is advocated. In this manner we can more confidently begin to interpret our study results.

## 4. Improving Adherence

Given the potential individual and global ramifications of noncompliance, interventions to promote compliance are needed. This is particularly important in the pediatric population where adherence to treatment regimens can be further influenced by involvement of family members, developmental capacity of the patient, and by unique psychosocial issues implicit at a given age [36–40]. Hence, a multifaceted approach tailored to the individual child/parent is needed to improve adherence [41–43]. Support from ancillary health care providers such as nurses, behavioral specialists, and pharmacists may increase adherence and should be used as needed when feasible [44–46]. We discuss approaches to enhance adherence for three age groups: infant/toddler/preschool (0–<5 yrs), young child (5–<11 yrs), and adolescent (11–<19 yrs).

In infants/toddlers/preschoolers, the caregiver is primarily responsible for the administration of the antibiotic. Enhancing patient/parent-doctor communication has a pivotal role in improving medication compliance [37, 47]. Here, the health care provider should assure that the caregiver has a solid understanding of the disease process, the benefits of the treatment regimen, and the risks of noncompliance. Dosing and timing of the medication prescribed should be discussed. As forgetfulness can be the main reason cited by caregivers for omitting antibiotic doses [48], short interval follow-up visits should be scheduled. This gives the healthcare provider an excellent opportunity to assess adherence in a nonjudgmental way, and reiterate the importance of following the treatment outlined. Also, the medication dose can be adjusted, as infants grow rapidly during this age.

Although there is a paucity of literature describing the most effective ways of improving adherence in young children, in general a combination of behavioral and educational strategies are most beneficial [49]. Education is typically targeted at the caregiver responsible for administering the medication, and involves written or verbal information about the child's illness, delineating reasons for treatment, and discussing potential consequences of poor adherence [49]. Other forms of education include informational videotapes, structured educational home visits by nurses or counselors, and group discussions [49–56]. When feasible, similar age appropriate verbal, written, and visual educational materials should be provided to the child. There is a wide spectrum of behavioral interventions for children which are simple and practical including positive reinforcement with rewards, diaries, stickers on calendars, goal setting, contracts, and linking medication intake with established daily routines, among others [49, 57–63]. The most common behavioral intervention is the token reinforcement system, where adherence to medication is rewarded by tokens or another form of reward [64–66]. It is important to inquire about medication/formulation preferences before prescribing prophylactic antibiotics (pill, capsule, or elixir form, and preferred flavor). Changing a medication to a more palatable form was shown to be effective in increasing adherence rates in children [67, 68]. A common complaint by parents/children with liquid nitrofurantoin is its poor taste. Perhaps the poor palatability may partly explain the distinctly low adherence rate of only 2% observed in children on nitrofurantoin prophylaxis for VUR [22]. Finally, the involvement of family members and schools can be valuable in improving adherence in children [35, 47]. 

While the underlying reasons for nonadherence in adolescents are complex and not fully understood, issues involving self-esteem, body image, coping mechanisms, and self motivation are contributing factors [38]. As children age and enter adolescence they become more autonomous and assume a greater responsibility for their own treatment [69]. In general, increased age is associated with lower adherence across many chronic pediatric illnesses including asthma, anxiety disorders, diabetes, and HIV [69–72]. Similar findings were noted by the Hensle et al. [21] and Copp et al. [22] groups with pediatric patients on CAP for VUR. This underscores the need for educational strategies targeted at the adolescent, as well as continuous parental oversight particularly as the child/adolescent is granted more autonomy. 

## 5. Conclusion

The goal of prophylactic antibiotics is to maintain urine sterility and prevent ascending urinary tract infections in children with vesicoureteral reflux until the condition resolves spontaneously or is surgically corrected. Nonadherence to prophylactic antibiotics among children is highly prevalent and may be associated with medication failure, unnecessary changes in treatment protocols, increased healthcare costs, and increased antibiotic resistance patterns. We must strive to identify risk factors for nonadherence, and implement behavioral/educational algorithms to improve adherence. Finally, in light of emerging data supporting the contention that prophylactic antibiotics may not be protective against UTIs, controlled studies that monitor and adequately account for adherence rates are needed. Until such studies are available, we cannot reliably determine whether antibiotic prophylaxis is effective in preventing urinary tract infection in children with vesicoureteral reflux.
